# Lost bones: differential diagnosis of acro-osteolysis seen by the pediatric rheumatologist

**DOI:** 10.1186/s12969-021-00596-0

**Published:** 2021-07-14

**Authors:** Elizaveta Limenis, Jennifer Stimec, Peter Kannu, Ronald M. Laxer

**Affiliations:** Division of Rheumatology, The Hospital for Sick Children, 555 University Ave, Toronto, ON M5G 1X8 Canada

**Keywords:** Acro-osteolysis, Pediatrics, Radiographs, Genetic disorders, Systemic sclerosis, Arthritis, Hyperparathyroidism, Neuropathy, Trauma, Ischemia

## Abstract

**Introduction:**

Acro-osteolysis is a radiographic finding which refers to bone resorption of the distal phalanges. Acro-osteolysis is associated with various conditions and its presence should prompt the clinician to search for the underlying etiology. The aim of this review is to discuss disorders with which acro-osteolysis is associated and their distinguishing features, with a focus on the pediatric population.

**Methods:**

A targeted literature review was performed using the term “acro-osteolysis” in combination with other key terms. The primary search results were supplemented using reference citations. Articles published prior to the year 2000 were included if they described additional associations not encountered in the more recent literature.

**Results:**

Genetic disorders (particularly primary hypertrophic osteoarthropathy and skeletal dysplasias) and rheumatic diseases (particularly psoriatic arthritis and systemic sclerosis) are the most frequently encountered conditions associated with acro-osteolysis in children. Hyperparathyroidism, neuropathy, local trauma and thermal injury, and spinal dysraphism should also be included in the differential diagnosis.

**Conclusion:**

Although acro-osteolysis is uncommon, its presence should prompt the clinician to consider a differential diagnosis based on clinical and radiographic features.

## Introduction

The term *acro-osteolysis* refers to bone resorption of the distal phalanges in the upper and lower extremities. Acro-osteolysis has been described in association with various disorders including genetic conditions, rheumatic diseases (psoriatic arthritis and systemic sclerosis in particular), hyperparathyroidism, severe neuropathy, digital ischemia, and trauma and other local factors. The underlying mechanism of bone resorption is largely unknown, although there is some evidence for vascular alterations leading to enhanced osteoclastic activity. Although acro-osteolysis may occur as an isolated idiopathic feature, its presence should prompt the clinician to obtain a targeted history and physical examination to search for the underlying etiology.

Plain radiographs are the gold standard for detecting acro-osteolysis. Two radiographic patterns of acro-osteolysis have been described: 1) resorption of the terminal tuft (more common), and 2) destruction of the proximal end of the distal phalanx causing a transverse osteolysis through the shaft. It has been suggested that the particular pattern of acro-osteolysis may be suggestive of the underlying etiology, with tuft resorption more commonly seen with systemic sclerosis, ischemia, hyperparathyroidism, and neurologic disorders, and destruction of the distal interphalangeal joint more commonly seen in inflammatory, particularly psoriatic, arthritis [1].

The literature to date has mostly described acro-osteolysis in the adult population. Although some of the conditions associated with acro-osteolysis, such as rheumatologic diseases, can span the age range, the approach to the differential diagnosis may be somewhat different in pediatric patients, with children being less likely to have environmental exposures and severe neuropathy, and more likely to have an underlying genetic disorder. The aim of this review is to describe the disorders with which acro-osteolysis is associated and their distinguishing features, with particular attention to those which are more commonly encountered in pediatrics.

## Methods

Relevant literature published since the year 2000 was screened, and original articles concerning studies in humans were included. A targeted literature review was performed in PubMed using the term *acro-osteolysis* alone and in various combination with the following terms: *imaging, radiographs, pediatrics, genetics, systemic sclerosis, psoriatic arthritis, mixed connective tissue disease, hyperparathyroidism, hypertrophic osteoarthropathy, neuropathy, trauma, ischemia, clubbing, Raynaud phenomenon, frostbite, cold, burns.* Titles, abstracts and full reports were screened for relevance and insight into the subject matter. The primary search results were supplemented using reference citations. Articles published prior to the year 2000 were included if they described additional associations with acro-osteolysis not encountered in the more recent literature. Case reports were included given the rarity of many of the conditions described.

### Genetic conditions

Acro-osteolysis has been well described in the setting of primary hypertrophic osteoarthropathy (PHO), also known as pachydermoperiostosis. This rare autosomal dominant or recessive condition is characterized by clubbing (near universal), periostitis, and acro-osteolysis of the terminal tufts. Arthralgias with or without arthritis secondary to periosteal inflammation are common. Dermatological findings include sweating of the palms (palmoplantar hyperhidrosis), skin thickening and furrowing of the forehead and scalp. Affected individuals may also report a history of persistent patent ductus arteriosus (PDA) and/or delayed closure of the fontanelles. To date, biallelic variants in 15-hydroxyprostaglandin dehydrogenase (HPGD) or solute carrier organic anion transporter family member 2A1 (SLCO2A1) have been associated with autosomal recessive forms of PHO. These gene mutations lead to chronically elevated prostaglandin E2 (PGE2), which is thought to stimulate osteoclastic activity and thereby lead to acro-osteolysis, and is also implicated in the pathogenesis of PDA [2]. An alternative proposed mechanism is that of PGE2 as a facilitator for vascular endothelial growth factor (VEGF), with the latter causing bone resorption by the same mechanisms described above [3].

**Fig. 1:**
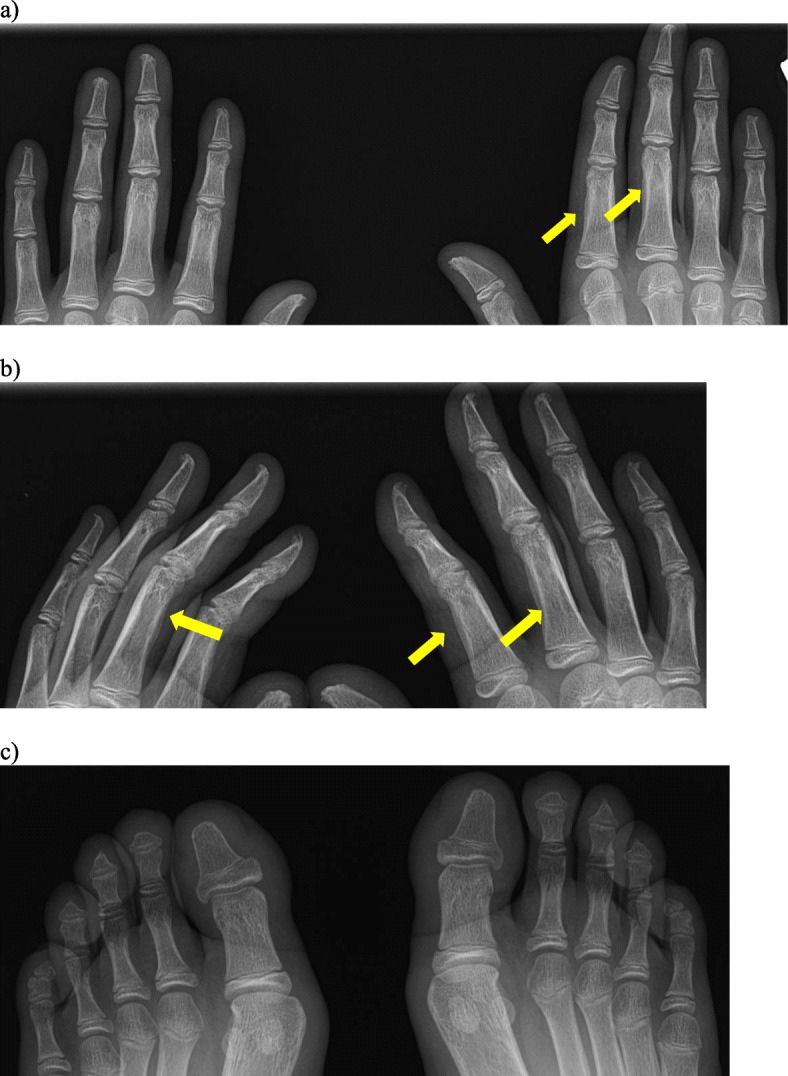
Frontal (**a**) and oblique (**b**) radiographs of the hands in a 14 year old male patient with primary hypertrophic osteoarthropathy demonstrate bilateral and symmetric tuft resorption and remodeling. Smooth periostitis involves the proximal phalangeal shafts (arrows). Frontal radiograph of the feet (**c**) shows smooth tapering of the distal tufts

We currently manage a 14 year old male patient affected by autosomal recessive PHO. He presented with polyarthralgias, marked palmoplantar hyperhidrosis, and clubbing of several toes as well as the second finger digits bilaterally. His extremity x-rays revealed acro-osteolysis and periostitis (Fig. 1). Genetic testing identified two different genetic variants in trans in the HPGD gene (c.418G > C; p.A140P and c.662 + 5_662 + 8del). Parental analysis confirmed each parent carried one variant.

It is important to note that hypertrophic osteoarthropathy (HO) can also occur in the setting of other underlying conditions, which should be included in the differential diagnosis. Secondary HO has most commonly been reported in adults with pulmonary disease, in particular thoracic tumors [3]. In children, historically, congenital heart disease and cystic fibrosis were two of the more common causes; it has been postulated that the frequency of HO secondary to these conditions has decreased over time as disease management and outcomes have improved [4]. A few case reports of HO have also been published in children secondary to osteosarcoma with pulmonary metastasis [5], hepatopulmonary syndrome [6], and biliary atresia [7].

A number of other single gene disorders can also cause acro-osteolysis in the setting of a syndromal presentation. A family history is useful in determining whether there is male to male transmission characteristic of an autosomal dominant inheritance pattern or a founder population and/or consanguinity which alternatively suggests a recessive pattern of inheritance. Autosomal dominant Hajdu-Cheney syndrome is an important differential diagnosis of acro-osteolysis. Hajdu-Cheney syndrome is caused by gain-of-function mutations in the neurogenic locus notch homolog protein 2 (NOTCH2) gene, which is implicated in skeletal development and bone remodeling. Other phenotypic findings include short stature, craniofacial anomalies, joint hypermobility, severe osteoporosis, and wormian bones [8]. Penttinen syndrome is an autosomal dominant premature aging syndrome caused by gain-of-function mutations in the platelet-derived growth factor receptor B (PDGFRB) gene. Phenotypic features include a prematurely aged appearance including lipoatrophy and epidermal and dermal atrophy, hypertrophic skin lesions that resemble scars, thin hair, proptosis, underdeveloped cheek bones and severe acro-osteolysis [9]. Autosomal dominant variants in the parathyroid hormone-like hormone (PTHLH) gene, which is important in mediating bone homeostasis, can result in a syndrome characterized by acro-osteolysis, cortical irregularity of long bones and metadiaphyseal enchondromata [10]. Autosomal dominant Warburg-Cinotti syndrome due to variants in discoidin domain receptor tyrosine kinase 2 (DDR2) is associated with progressive corneal vascularization, keloid formation, chronic skin ulcers, wasting of subcutaneous tissue, flexion contractures of the fingers, and acro-osteolysis [11]. Autosomal dominant Singleton-Merten syndrome is an interferonopathy characterized by variable expression of aortic calcification, dental anomalies, osteopenia and acro-osteolysis, and to a lesser degree glaucoma, psoriasis, muscle weakness and joint laxity [12]. Acro-osteolysis has also been described as a rare manifestation of lysosome storage disorders [13], and in the setting of epidermolysis bullosa [14]. Of interest, acro-osteolysis was described in an adult patient with symphalangism, with several autosomal dominant variants in the NOG gene (noggin) [15]. Acro-osteolysis has never previously been described in NOG-related syndromes and it is thus unclear if this is an additional phenotypic finding or an unrelated manifestation.

If the family history is suggestive of an autosomal recessive inheritance pattern, a different group of single gene disorders enter the differential diagnosis. Multicentric Osteolysis Nodulosis and Arthropathy (MONA), also termed Torg-Winchester syndrome, is an autosomal recessive skeletal dysplasia cause by variants in the matrix metalloproteinase-2 (MMP2) gene. MONA is characterized by a coarse facial appearance, progressive arthropathy, diffuse osteopenia, and characteristic subcutaneous nodules. Radiographically, lysis of carpal and tarsal bones is seen, often extending to the metacarpals, metatarsals and phalanges [16]. Recessive variants in the LMNA gene (lamin A/C) were recently described in a Saudi male patient from a consanguineous family with scleroderma-like skin thickening, joint contractures restricting joint mobility, poikiloderma and acro-osteolysis [17]. Another recessive condition which includes acro-osteolysis is Haim-Munk syndrome. This condition is characterized by palmoplantar hyperkeratosis, psoriasis-like rash, periodontitis, and acro-osteolysis [18].

### Rheumatic diseases

#### Psoriatic arthritis

In the pediatric population, psoriatic juvenile idiopathic arthritis (jPsA) constitutes one of the subtypes of juvenile idiopathic arthritis (JIA). Musculoskeletal involvement in jPsA may include arthritis, dactylitis, enthesitis and axial disease. Involvement of the distal interphalangeal (DIP) joints is more commonly associated with jPsA, and is rarely seen in other subtypes of JIA. jPsA can lead to severe joint damage and deformity. Radiographic features of psoriatic arthritis (PsA) include joint space narrowing and erosions, periostitis, spur formation, ankylosis, and osteolysis including acro-osteolysis [19]. Both patterns of acro-osteolysis can be seen – that of terminal tuft resorption and destruction of the proximal end of the distal phalanx, the latter being rather unique to PsA [1]. Several case reports have described acro-osteolysis in association with PsA, including a case of a middle-aged male with severe psoriatic onychodystrophy and arthritis of the first metatarsophalangeal (MTP) joints [20].

**Fig. 2:**
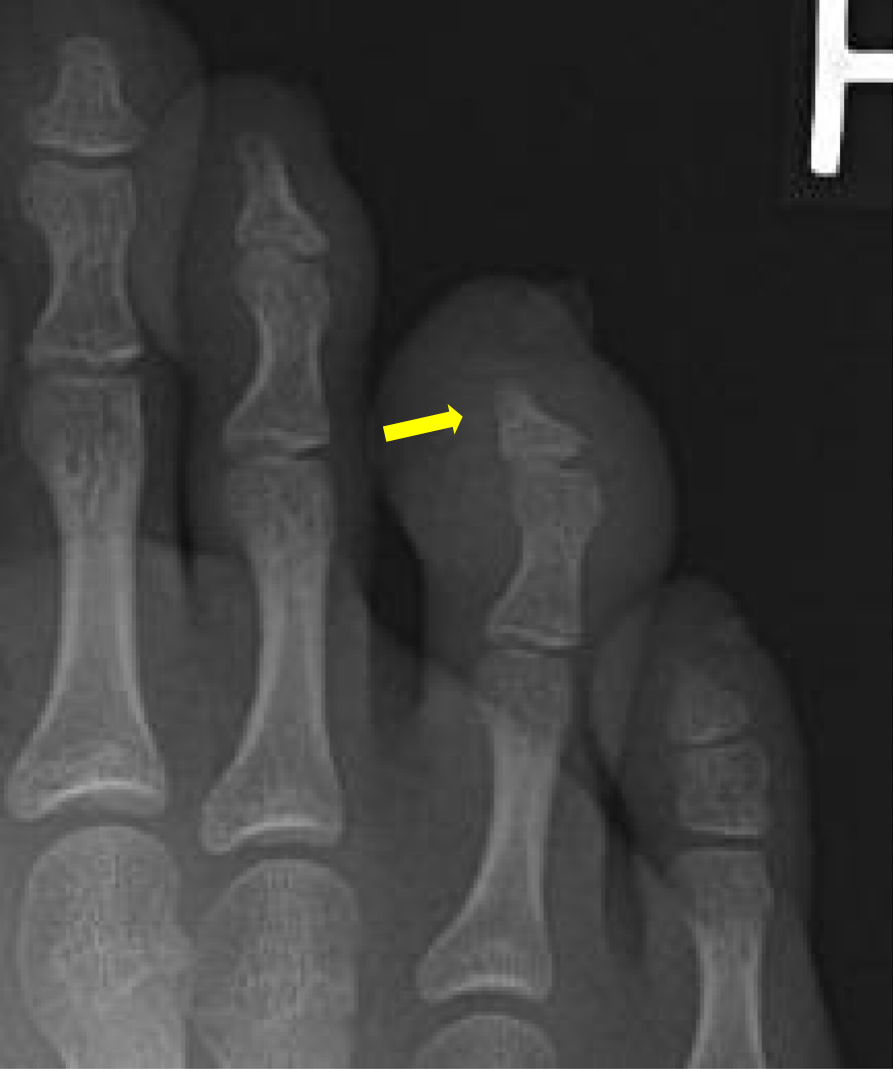
Frontal radiograph of the toes in a 14 year old female patient with juvenile psoriatic arthritis demonstrates focal soft tissue swelling of the fourth digit, with tapering of the distal phalanx and resorption of the distal tuft cortex

We currently care for a 14 year old female patient with jPsA and acro-osteolysis of one toe (Fig. 2). She presented with a history of psoriasis and subacute onset of pain in the affected toe limiting ambulation.

It is worth noting that acro-osteolysis has been reported in association with psoriasis alone, in the absence of arthropathy. Review of the literature revealed a case of a middle-aged woman with psoriasis vulgaris and onycholysis, with severe osteolysis of the distal phalanx of 3 digits [21], and a case of an elderly man with palmoplantar psoriasis and symmetrical shortening of the distal phalanges, loss of the nails on most digits, and terminal tuft resorption in all finger digits on radiographic imaging [22].

#### Systemic sclerosis

While systemic sclerosis (SSc) is much more commonly diagnosed in adult patients, a minority of SSc patients present in childhood, most commonly with Raynaud phenomenon and/or skin changes [23]. Radiographic features of SSc include joint space narrowing, erosion, osteopenia, flexion contracture, calcinosis and acro-osteolysis [24].

Acro-osteolysis is a well-recognized feature of SSc, reported to occur in 6–65% of patients [25], 22–33% in more recently published cohorts [24, 26, 27]. When present together with Raynaud phenomenon, acro-osteolysis is highly suggestive of SSc. A large study comparing hand radiographs in patients with SSc with those of controls found that acro-osteolysis and calcinosis were the most common radiologic features in SSc patients and were almost completely absent in controls [27]. The pattern of acro-osteolysis in SSc is that of terminal tuft resorption, typically beginning at the palmar aspect of the bone with progressive sharpening of the distal phalanx. The course is variable, with osteolysis stabilizing in some patients and progressing towards complete loss of the distal phalanges in others. Osteolysis in SSc is associated with more severe disease, including digital ulceration [25–28], pulmonary arterial hypertension (PAH) [24], severe digital ischemia [26–28], severe calcinosis [24, 28, 29], and longer disease duration [27, 29]. Radiographic progression of flexion contractures has also been reported to be significantly higher in patients with acro-osteolysis [28]. However, at least one case has been described of severe “catastrophic” acro-osteolysis (including nearly complete resorption of all distal and middle phalanges of the fingers) in a patient with SSc in the absence of digital ulceration, significant calcinosis and PAH, suggesting that other factors are likely to be involved [29].

**Fig. 3:**
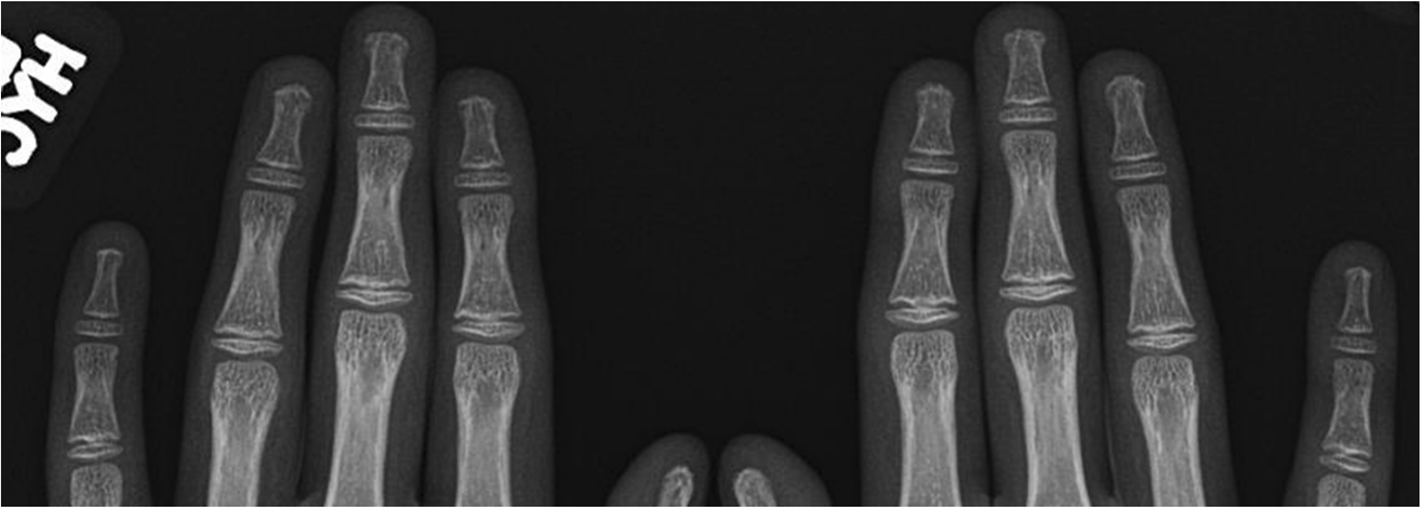
Frontal radiographs of the hands in a 13 year old male patient with systemic sclerosis show tuft resorption consistent with acro-osteolysis. No soft tissue calcifications present

The authors of this review follow a 13 year old male patient with SSc who presented with longstanding skin thickening and Raynaud phenomenon, without signs of severe digital ischemia, and was found to have terminal tuft resorption of the distal phalanges in the hands (Fig. 3).

Although the pathogenesis of acro-osteolysis in SSc is not well established, it is postulated that the most important underlying mechanism involves vascular alterations and reduced capillary density impairing tissue oxygenation. Given that angiogenesis is important to maintaining bone homeostasis, it is thought that impaired vascular supply may lead to enhanced osteoclastic activity and thereby bone resorption. It has been shown that tissue hypoxia leads to increased levels of hypoxia-inducible factor-1α (HIF-1α). The HIF pathways regulate pro-angiogenic genes, and one consequence of increased HIF-1α is increased levels of VEGF. There is evidence that increased VEGF leads to increased osteoclastogenesis and defective angiogenesis in SSc [25].

Clinically, this proposed mechanism is supported by the correlation between severe digital ischemia with severe acro-osteolysis. In a study of 101 patients with SSc, a grading scale for acro-osteolysis ranging from 0 to 4 was developed and applied to each digit, with 0 defined by normal bone structure and 4 defined by complete terminal tuft resorption with severe pencilling. Of the 68 patients with no or minimal acro-osteolysis, 29% had severe digital ischemia, compared to 76% of the 33 patients with moderate or severe acro-osteolysis (*p* < 0.001) [26]. Other studies evaluating radiographic hand damage in SSc have also found a strong correlation between acro-osteolysis and disease severity, including severe vascular involvement [27, 28], subcutaneous calcification [28], and digital ulcers [28]. Tapering of the fingers in SSc is also thought to be due to growth arrest secondary to chronic digital ischemia [30]. These findings reinforce the probable mechanistic link between severe digital ischemia and acro-osteolysis, and suggest that treatment should primarily target SSc vasculopathy.

In addition to vascular impairment, the association of acro-osteolysis with secondary hyperparathyroidism suggests another potential mechanism for the development of bone resorption at the terminal tufts, including in the context of SSc. A study evaluating 60 Mediterranean patients with SSc reported the presence of acro-osteolysis in 70% and vitamin D deficiency in 46% of patients. The study found a significant correlation between acro-osteolysis and elevated parathyroid hormone (PTH) levels, suggesting that secondary hyperparathyroidism from vitamin D deficiency may reflect silent malabsorption in SSc patients and may be an important contributor to acro-osteolysis [31]. Additional studies are needed to evaluate this relationship further.

#### Other rheumatic diseases

Acro-osteolysis has rarely been described in other systemic rheumatic diseases, for instance mixed connective tissue disease (MCTD) in which features of SSc (in particular Raynaud phenomenon and swollen digits) may be present with overlapping features of systemic lupus erythematosus (SLE) and/or myositis. One study comparing articular disease in MCTD and SLE patients found that radiographic acro-osteolysis was significantly more common in the MCTD group, with no difference in erosive joint disease [32]. However, at least one case report has been described of a patient with SLE developing acro-osteolysis of the terminal tufts of several fingers and toes in the absence of Raynaud phenomenon, nailfold capillary changes, digital ulceration and other features suggesting digital ischemia or overlap with SSc [33].

Isolated case reports have been published regarding acro-osteolysis in the context of other rheumatic diseases. One case report describes a man with granulomatosis with polyangiitis (GPA) who presented with acro-osteolysis, mononeuritis multiplex, and asymptomatic lung disease detected on imaging [34]. Another report describes an elderly man with primary Raynaud phenomenon (and exclusion of systemic rheumatic disease) who developed acro-osteolysis of almost all terminal phalanges of the fingers [35]. A review from 1986 notes reports of acro-osteolytic bony changes in the context of sarcoidosis, osteoarthritis, and arthritis associated with biliary cirrhosis [1]. Sarcoid-associated acro-osteolysis has also been described in a more recent case of a 44 year old woman with biopsy-proven sarcoidosis [36].

### Hyperparathyroidism

Acro-osteolysis, typically in the context of more widespread osteolysis, has been reported in patients with secondary hyperparathyroidism from chronic kidney disease [37, 38]. As described above, it is plausible that hyperparathyroidism secondary to vitamin D deficiency may play a role in the development of acro-osteolysis in other conditions.

**Fig. 4:**
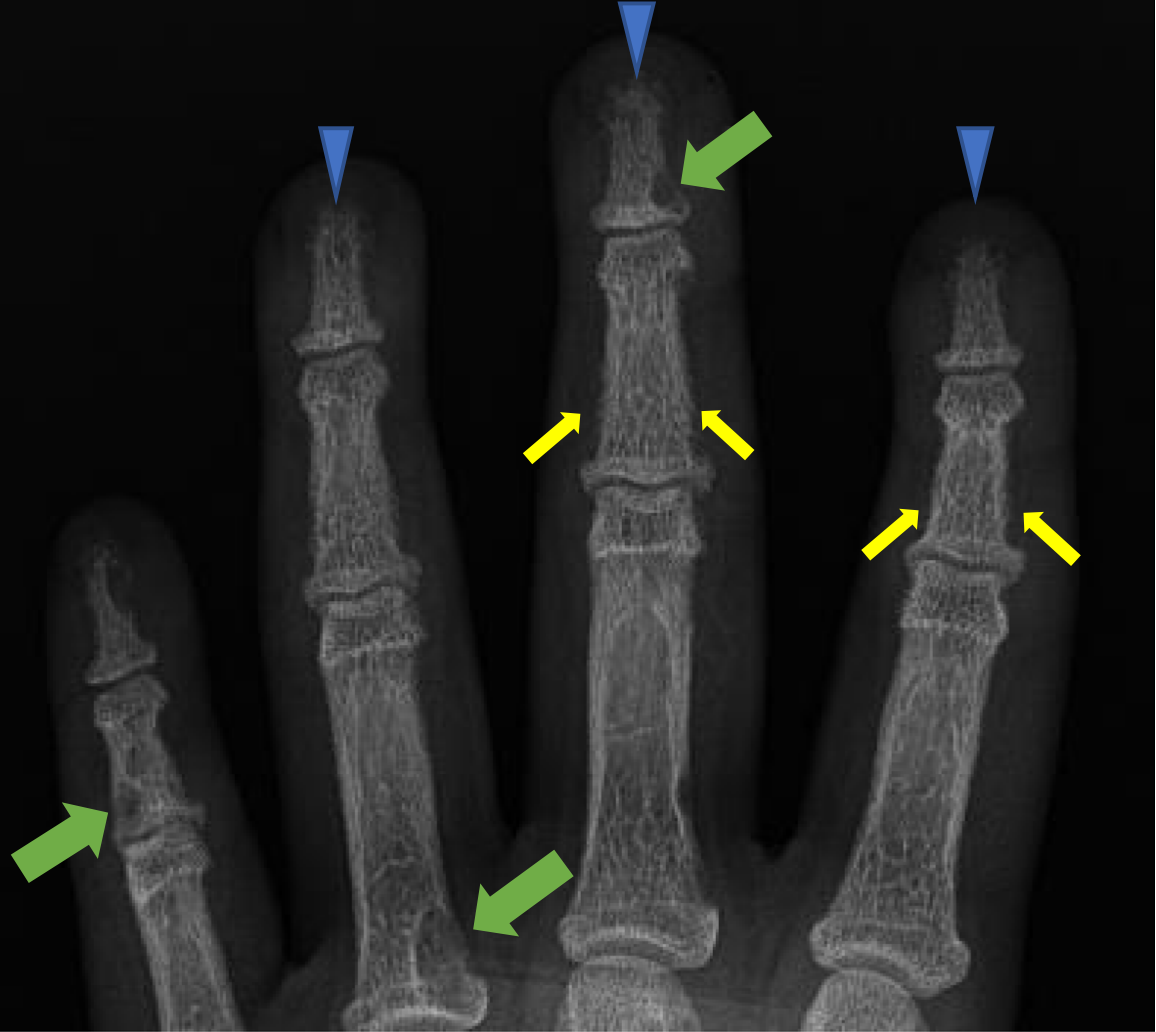
Frontal radiograph of the left hand in a 14 year old male patient with hyperparathyroidism demonstrates acro-osteolysis (arrowheads), subperiosteal resorption at the middle phalanges (small arrows), brown tumors (large arrows) and diffuse osteopenia

A hand radiograph of a 14 year old male patient with hyperparathyroidism followed at our institution revealed acro-osteolysis, subperiosteal resorption, diffuse osteopenia and brown tumors (Fig. 4).

### Neuropathy

Acro-osteolysis has rarely been described in adult patients in the setting of severe neuropathy secondary to carpal tunnel syndrome and diabetes mellitus. There are several case reports of severe carpal tunnel syndrome leading to acro-osteolysis, nail dystrophy and digit ulceration occurring in the median nerve distribution, either unilaterally or bilaterally [39, 40]. There is also one case report of acro-osteolysis and superficial ulceration of the distal phalanges in a 45 year old woman with a 16-year history of poorly controlled diabetes mellitus type 1 [41]. The authors propose a shared mechanism to that in carpal tunnel syndrome, with sensory neuropathy leading to acro-osteolysis in both conditions. Acro-osteolysis secondary to neuropathy in the setting of leprosy has also been described in two unrelated patients [42]. Lastly, there are several older reports of families with hereditary sensory neuropathies leading to acro-osteolysis and severe ulcerative and mutilating disease [43, 44]. One such example is congenital insensitivity to pain with anhidrosis (CIPA), a very rare hereditary sensory autonomic neuropathy characterized by developmental delay, joint deformities, fractures, dislocations, osteomyelitis, avascular necrosis and acro-osteolysis [45].

### Miscellaneous

Historically, occupational acro-osteolysis was reported in association with polyvinyl chloride, presenting with Raynaud phenomenon and SSc-like skin changes [46]. This is largely a disease of the past, with no new cases reported in the last two decades.

Acro-osteolysis has been reported secondary to local trauma and thermal injury. It has been described in the setting of repetitive trauma in two adolescent ballet dancers, and an adult surfer [47, 48]. Reports of frostbite leading to acro-osteolysis suggest that it tends to occur months after the injury and typically spares the thumb. The mechanism is thought to be related to vascular insufficiency or direct cellular injury, and may involve superimposed infection [1]. A recent case has been reported of brachyonychia and nail dystrophy 1 year following severe cold exposure, followed by seasonal chilblains, clubbing, disruption of nailfold capillaries and acro-osteolysis of the terminal tufts on imaging, with an otherwise negative systemic work-up [49]. Severe burns are also a rare cause of acro-osteolysis. A recent case of ankylosis of interphalangeal joints, flexion contracture deformity and terminal tuft resorption was reported in the hand of a woman who was recovering from a burn injury [50].

Lastly, unilateral lower extremity acro-osteolysis has been reported as the presenting feature of occult spinal dysraphism in several patients including cases in children [51, 52]. Whereas most causes of acro-osteolysis outlined above would manifest with bilateral involvement, unilateral acro-osteolysis should prompt the clinician to consider local trauma and spinal dysraphism in the differential diagnosis.

## Conclusions

Although acro-osteolysis is uncommon, its presence should prompt the clinician to consider a differential diagnosis based on clinical features and the radiographic pattern (Table 1). Genetic disorders are especially relevant in the pediatric setting, in particular PHO and those within the skeletal dysplasia family. Of the rheumatic diseases, acro-osteolysis may occur in a number of the systemic disorders but has been best described in the setting of PsA and SSc, both of which may occur in childhood and present with other manifestations of those conditions. Other systemic disorders to consider include hyperparathyroidism and secondary HO in the setting of pulmonary or hepatic disease. Conditions that lead to severe sensory neuropathy may be considered, although these are less likely to be encountered in the pediatric setting. Lastly, unilateral acro-osteolysis should prompt the clinician to consider spinal dysraphism if presenting in a lower extremity, as well as local factors such as trauma or thermal injury.

**Table 1:** Distinguishing clinical features and radiographic appearance of conditions that may present with acro-osteolysis in children

Differential diagnosis of acro-osteolysis	Distinguishing Clinical Features	Radiographic Appearance
Genetic Disorders		**Terminal tuft resorption**
*Primary hypertrophic osteoarthropathy (PHO)*	Clubbing, hyperhidrosis *Consider secondary HO	Tuft resorption involving toes then fingers, periostitis
*Hajdu-Cheney Syndrome*	Short stature, craniofacial abnormalities, severe osteoporosis, bone deformities	Progressive diffuse bone resorption
*Skeletal dysplasias and related disorders*	Bone abnormalities	Cortical bone irregularities, osteopenia
*Laminopathies*	Involvement of skin, fat, muscle	
Rheumatic Diseases		
*Psoriatic arthritis (PsA)*	Psoriatic skin and/or nail changes, arthritis (predilection for distal interphalangeal joint involvement)	**Destruction of interphalangeal joint** (joint space narrowing, erosions), terminal tuft resorption, periostitis, spur formation, ankylosis
*Systemic sclerosis (SSc)*	Raynaud phenomenon, skin thickening, flexion contractures	**Terminal tuft resorption,** joint space narrowing, erosion, osteopenia, flexion contractures, calcinosis
*Others*	Systemic symptoms	
Hyperparathyroidism	1Underlying conditions leading to secondary hyperparathyroidism (e.g. Vitamin D deficiency, renal disease)	**Terminal tuft resorption,** diffuse osteolysis, brown tumors (lytic lesions)
	Symptoms of hypercalcemia in primary hyperparathyroidism	
Neuropathy	Underlying conditions (e.g. diabetes mellitus, carpal tunnel syndrome), hereditary forms	**Terminal tuft resorption**
Miscellaneous	Unilateral involvement	**Terminal tuft resorption**
*Local factors: Repetitive trauma, thermal injury*	Exposure on clinical history	
*Occult spinal dysraphism*	Lower extremity involvement	

## Data Availability

Data sharing is not applicable to this article as no datasets were generated or analysed during the current study.

## Abbreviations

PHO: Primary hypertrophic osteoarthropathy
PDA: Patent ductus arteriosus
HPGD: 15-Hydroxyprostaglandin dehydrogenase
SLCO2A1: Solute carrier organic anion transporter family member 2A1
PGE2: Prostaglandin E2
VEGF: Vascular endothelial growth factor
HO: Hypertrophic osteoarthropathy
NOTCH2: Neurogenic locus notch homolog protein 2
PDGFRB: Platelet-derived growth factor receptor B
PTHLH: Parathyroid hormone-like hormone
DDR2: discoidin domain receptor tyrosine kinase 2
MONA: multicentric osteolysis nodulosis and arthropathy
MMP2: Matrix metalloproteinase-2
jPsA: Psoriatic juvenile idiopathic arthritis
JIA: Juvenile idiopathic arthritis
DIP: Distal interphalangeal
PsA: Psoriatic arthritis
MTP: Metatarsophalangeal
SSc: Systemic sclerosis
PAH: Pulmonary arterial hypertension
HIF-1α: Hypoxia-inducible factor-1α
PTH: Parathyroid hormone
MCTD: Mixed connective tissue disease
SLE: Systemic lupus erythematosus
GPA: Granulomatosis with polyangiitis
CIPA: Congenital insensitivity to pain and anhidrosis
